# Fluorometric Measurement of Calmodulin-Dependent Peptide–Protein Interactions Using Dansylated Calmodulin

**DOI:** 10.21769/BioProtoc.4963

**Published:** 2024-04-05

**Authors:** Eider Nuñez, Arantza Muguruza-Montero, Sara M. Alicante, Alvaro Villarroel

**Affiliations:** Instituto Biofisika, CSIC-UPV/EHU, Leioa, Spain

**Keywords:** Dansyl-Calmodulin, CaM-target, Steady-state fluorescence spectroscopy, Calcium dependency, Dansyl-chloride, Peptide, Protein

## Abstract

The assessment of peptide–protein interactions is a pivotal aspect of studying the functionality and mechanisms of various bioactive peptides. In this context, it is essential to employ methods that meet specific criteria, including sensitivity, biocompatibility, versatility, simplicity, and the ability to offer real-time monitoring. In cellular contexts, only a few proteins naturally possess inherent fluorescence, specifically those containing aromatic amino acids, particularly tryptophan. Nonetheless, by covalently attaching fluorescent markers, almost all proteins can be modified for monitoring purposes. Among the early extrinsic fluorescent probes designed for this task, dansyl chloride (DNSC) is a notable option due to its versatile nature and reliable performance. DNSC has been the primary choice as a fluorogenic derivatizing reagent for analyzing amino acids in proteins and peptides for an extended period of time. In our work, we have effectively utilized the distinctive properties of dansylated-calmodulin (D-CaM) for monitoring the interaction dynamics between proteins and peptides, particularly in the context of their association with calmodulin (CaM), a calcium-dependent regulatory protein. This technique not only enables us to scrutinize the affinity of diverse ligands but also sheds light on the intricate role played by calcium in these interactions.

Key features

• Dynamic fluorescence and real-time monitoring: dansyl-modified CaM enables sensitive, real-time fluorescence, providing valuable insights into the dynamics of molecular interactions and ligand binding.

• Selective interaction and stable fluorescent adducts: DNSC selectively interacts with primary amino groups, ensuring specific detection and forming stable fluorescent sulfonamide adducts.

• Versatility in research and ease of identification: D-CaM is a versatile tool in biological research, facilitating identification, precise quantification, and drug assessment for therapeutic development.

• Sensitivity to surrounding alterations: D-CaM exhibits sensitivity to its surroundings, particularly ligand-induced changes, offering subtle insights into molecular interactions and environmental influences.

**Graphical overview**


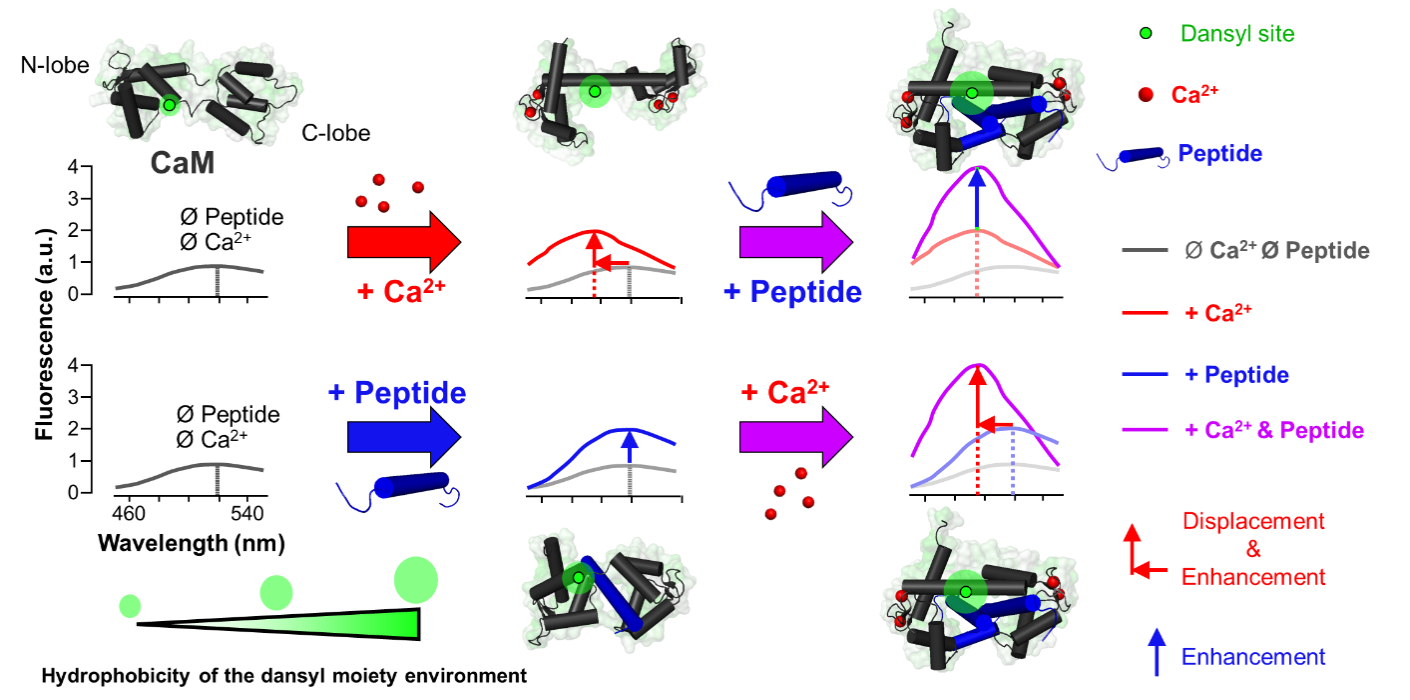


**Fluorescence emission profiles of dansylated-calmodulin (D-CaM) in different states.** Fluorescence emission spectra of D-CaM upon excitation at 320 nm are depicted. Conditions include apo-D-CaM (gray), holo-D-CaM (red), apo-D-CaM bound to peptide (blue), and holo-D-CaM bound to peptide (purple). Corresponding structural representations of D-CaM next to each condition are superimposed on the respective spectra along with the hydrophobicity of the dansyl environment, which increases upon binding of peptide or Ca^2+^ to D-CaM. Upon peptide binding to D-CaM, there is an enhancement in the fluorescent intensity of the spectra; upon Ca^2+^ binding, there is an enhancement of the intensity and a leftward shift of the spectra.

## Background

Calmodulin (CaM), a pivotal Ca^2+^-binding protein, intricately regulates essential biological functions by binding to the cation, thereby exerting meticulous control over an array of effector proteins. This interaction induces conformational changes in CaM, critically influencing cellular processes such as muscle contraction and neurotransmitter release, as exhaustively elucidated by Chin and Means [1] and Rhoads and Friedberg [2].

The trajectory of unraveling CaM's multifaceted role spans decades, commencing in the 1970s with the identification of cyclic nucleotide phosphodiesterase as one of the initial proteins binding to CaM, as underscored by Rasmussen et al. [3]. Subsequently, Klee and Vanaman's seminal work in 1982 laid the foundational understanding of CaM's centrality in cellular signal transduction. The ongoing delineation of over 300 target peptides for CaM, meticulously documented by Klee and Vanaman [4], accentuates its indispensability in diverse cellular processes.

Technological advancements, such as dansylation [5] and fluorogenesis [6], have significantly contributed to the precise identification and characterization of CaM targets, thereby enhancing our understanding of fundamental cellular processes. Among these tools, dansyl-CaM (D-CaM), a derivative of CaM conjugated with dansyl chloride (DNSC) [5-(dimethylamino)naphthalene-1-sulfonyl chloride], emerges as a distinctive and powerful instrument for analyzing interactions with peptides and proteins. DNSC specifically interacts with primary amino groups, forming stable blue or blue-green fluorescent sulfonamide adducts with aliphatic and aromatic amines (Tyr, Phe, Trp, etc.). The incorporation of dansyl into CaM enables the sensitive detection and thorough examination of this modified protein using fluorescence-based techniques.

High-resolution structural scrutiny of apo-CaM and holo-CaM has unveiled intricate Ca^2+^-induced structural changes, laying bare hydrophobic interfaces, aligning with the observations of Chin and Means [1] and Rhoads and Friedberg [2]. Structural analyses of CaM–peptide complexes reveal a commonality in interaction patterns, particularly in the hydrophobic domains of the CaM protein, resembling interactions observed with classical proteins and inhibitors [2]. Significantly, these peptides manifest a positively charged amphiphilic alpha-helical structure, irrespective of their amino acid sequences, as expounded upon by O’Neil and DeGrado [7]. The observed high affinity (Kd) of these peptides, falling in the range of 10^-9^–10^-12^ M, positions them as putative bioactive entities, exceeding the affinity of traditional organic CaM inhibitors (Kd ~10^-3^ M), as demonstrated by Chen et al. [8] and Peersen et al. [9].

Nevertheless, the conventional understanding of CaM function encounters challenges with the discovery of proteins preferentially binding to apo-CaM, influencing their Ca^2+ ^affinity, as exemplified by Smith et al. [10]. Additionally, CaM-binding partners exhibit minimal or no sequence similarity, posing a conundrum for attempts at structural categorization.

In navigating this intricate terrain, the elucidated method emerges as a potent tool, facilitating expeditious, cost-effective, and reliable identification of CaM targets even in instances of deviation from canonical binding patterns. This approach not only sheds light on diverse interactions involving D-CaM but also imparts invaluable insights into the nuanced relationships between proteins within the dynamic realm of CaM.

## Materials and reagents

**Biological materials**

1. Calmodulin in pET14b plasmid or other bacterial protein expression vector
2. Peptides from Proteogenix (https://www.proteogenix.science/) or another source
  Peptides exhibiting sequences, structures, or functions favorable for CaM binding are recommended, with an advised minimum length of 5 and a maximum of 50 amino acids to avoid potential challenges in purity and yield. See Note 1.
3. *E. coli* BL21 DE3 (Sigma-Aldrich, catalog number: 69450-M) (other strains may work as well)

**Reagents**

1. Dansyl chloride (DNSC) (Merck, Supelco, catalog number: 03641)
2. Sephadex G-25 (Merck, Sigma-Aldrich, catalog number: S5772)
3. Sepharose CL-4B (Merck, Sigma-Aldrich, catalog number: 4B200)
4. Base Trizma (Tris) (Merck, Sigma-Aldrich, catalog number: T1503)
5. HEPES (Thermo Fisher, Thermo Scientific Chemicals, catalog number: J16926.A1)
6. Potassium chloride (KCl) (Merck, Sigma-Aldrich, catalog number: P9541)
7. Ethyleneglycol-bis(β-aminoethyl)-N,N,N,N-tetraacetic acid (EGTA) (Merck, Millipore, catalog number: 324626)
8. Calcium chloride dihydrate (CaCl_2_) (Merck, Millipore, catalog number: 208291)
9. Sodium chloride (NaCl) (Merck, Sigma-Aldrich, catalog number: S3014)
10. 30% Acrylamide/Bis solution, 37.5:1 (Bio-Rad, catalog number: 1610158)
11. Sodium dodecyl sulfate (SDS) (Merck, Sigma-Aldrich, catalog number: 436143)
12. Ammonium persulfate (APS) (Merck, Sigma-Aldrich, catalog number: A3678)
13. Tetrametiletilendiamina (TEMED) (Thermo Fisher, catalog number: 17919)
14. Isopropanol (Merck, Sigma-Aldrich, catalog number: I9516)
15. Coomassie Brilliant Blue R-250 powder (CBB R-250) (Bio-Rad, catalog number: 1610400)
16. Methanol (Merck, Sigma-Aldrich, catalog number: 1060351000)
17. Acetic acid (glacial) 100% (Merck, Supelco, catalog number: 100066)
18. Phenyl-Sepharose CL-4B (Merck, Cytiva, catalog number: GE17-0150-01)
19. Dimethyl Sulfoxide (DMSO) (Merck, Sigma-Aldrich, catalog number: D2650)
20. N,N-Dimethylformamide (DMF) (Merck, Supelco, catalog number: DX1730)
21. Acetone (Merck, Sigma-Aldrich, catalog number: 179124)
22. Ethanol (Merck, Sigma-Aldrich, catalog number: 34852-M)
23. Glycerol (Merck, Sigma-Aldrich, catalog number: G5516)
24. Nitrocellulose membrane (Merck, Cytiva, catalog number: GE10600001)
25. Bradford (Merck, Supelco, catalog number: B6916)
26. Ponceau Red (Ponceau 4R) (Merck, Supelco, catalog number: 18137)
27. Trifluoroacetic acid (TFA) (Merck, Sigma-Aldrich, catalog number: 302031)
28. Ammonium hydroxide solution (NH_4_OH) (Merck, Sigma-Aldrich, catalog number: 221228)
29. Acetonitrile (Merck, Sigma-Aldrich, catalog number: 34851)
30. LB broth (Lennox) (Merck, Sigma-Aldrich, catalog number: L3022)
31. Isopropyl β-D-1-thiogalactopyranoside (IPTG) (Merck, Sigma-Aldrich, catalog number: I5502)
32. Phenylmethanesulfonyl fluoride (PMSF) (Merck, Roche, catalog number: 10837091001)

**Solutions**

1. Dansylation buffer (D buffer) (see Recipes)
2. Lysis buffer (L buffer) (see Recipes)
3. Equilibration buffer (CQ buffer) (see Recipes)
4. Wash buffer (CW buffer) (see Recipes)
5. High salt wash buffer (CHSW buffer) (see Recipes)
6. Elution buffer (CE buffer) (see Recipes)
7. Fluorescence buffer (F buffer) (see Recipes)
8. Calcium buffer (Ca buffer) (see Recipes)
9. APS 10% (see Recipes)
10. SDS 10% (see Recipes)
11. Tris-HCl 1.5 M, pH 8.8 (see Recipes)
12. Tris-HCl 1 M, pH 6.8 (see Recipes)
13. 15% Acrylamide electrophoresis gel RESOLVING (see Recipes)
14. Stacking acrylamide gel (see Recipes)
15. Running buffer 10× (see Recipes)
16. Loading buffer 5× (see Recipes)
17. Coomassie Blue (see Recipes)
18. Fast De-staining solution (see Recipes)
19. De-staining solution (see Recipes)
20. Ampicillin 100 μg/mL (see Recipes)
21. Ponceau Red (see Recipes)

**Recipes**

1. **Dansylation buffer (D buffer)**
  **Table:** 

Reagent	Final concentration	Quantity
CaCl_2_ (1 M)	20 mM	2 mL
Tris-HCl (1 M, pH 8.5)	100 mM	10 mL
H_2_O	n/a	88 mL
Total	n/a	100 mL
2. **Lysis buffer (L buffer)**
  **Table:** 

Reagent	Final concentration	Quantity
EDTA (1 M)	2 mM	0.2 mL
Tris-HCl (1 M, pH 7.5)	50 mM	5 mL
PMSF (0.1 M)	2 mM	2 mL
H_2_O	n/a	83.8 mL
Total	n/a	100 mL
3. **Equilibration buffer (Q buffer)**
  **Table:** 

Reagent	Final concentration	Quantity
CaCl_2_ (1 M)	5 mM	0.5 mL
Tris-HCl (1 M, pH 7.5)	50 mM	5 mL
NaCl (1 M)	100 mM	10 mL
H_2_O	n/a	84.5 mL
Total	n/a	100 mL
4. **Wash buffer (W buffer)**
  **Table:** 

Reagent	Final concentration	Quantity
CaCl_2_ (1 M)	0.1 mM	10 µL
Tris-HCl (1 M, pH 7.5)	50 mM	5 mL
NaCl (1 M)	100 mM	10 mL
H_2_O	n/a	84.90 mL
Total	n/a	100 mL
5. **High salt wash buffer (CHSW buffer)**
  **Table:** 

Reagent	Final concentration	Quantity
CaCl_2_ (1 M)	0.1 mM	10 µL
Tris-HCl (1 M, pH 7.5)	50 mM	5 mL
NaCl (1 M)	500 mM	50 mL
H_2_O	n/a	44.90 mL
Total	n/a	100 mL
6. **Elution buffer (E buffer)**
  **Table:** 

Reagent	Final concentration	Quantity
EGTA (1 M)	1 mM	100 µL
Tris-HCl (1 M, pH 7.5)	50 mM	5 mL
Total	n/a	94.9 mL
7. **Fluorescence buffer (F buffer)**
  **Table:** 

Reagent	Final concentration	Quantity
KCl (1 M)	120 mM	12 mL
HEPES (1 M, pH 7.4)	50 mM	5 mL
NaCl (1 M)	5 mM	0.5 mL
EGTA (1 M)	5 mM	0.5 mL
H_2_O	n/a	82 mL
Total	n/a	100 mL
  See Note 2.
8. **Calcium buffer (Ca buffer)**
  **Table:** 

Reagent	Final concentration	Quantity
KCl (1 M)	120 mM	12 mL
HEPES (1 M, pH 7.4)	50 mM	5 mL
NaCl (1 M)	5 mM	0.5 mL
EGTA (1 M)	5 mM	0.5 mL
CaCl_2_ (1 M)	20 mM	2 mL
H_2_O	n/a	80 mL
Total	n/a	100 mL
  See Note 3.
9. **APS 10%**
  **Table:** 

Reagent	Final concentration	Quantity
APS	10%	1 g
H_2_O	n/a	10 mL
10. **SDS 10%**
  **Table:** 

Reagent	Final concentration	Quantity
SDS	10%	1 g
H_2_O	n/a	10 mL
11. **Tris-HCl pH 8.8 1.5 M**
  **Table:** 

Reagent	Final concentration	Quantity
Tris	1.5 M	36.3 g
H_2_O	n/a	200 mL
  *Note 4
12. **Tris-HCl 1 M pH 6.8**
  **Table:** 

Reagent	Final concentration	Quantity
Tris	1 M	12.1 g
H_2_O	n/a	100 mL
  See Note 5.
13. **15% Acrylamide electrophoresis gel RESOLVING (for one gel)**
  **Table:** 

Reagent	Final concentration	Quantity
Acrylamide	15%	2.5 mL
Tris 1.5 M, pH 8.8	390 mM	1.3 mL
10% SDS	0.1%	50 µL
10% APS	0.1%	50 µL
TEMED	0.004%	2 µL
H_2_O	n/a	1.2 mL
Total	n/a	5 mL
14. **Electrophoresis gel STACKING (for one gel)**
  **Table:** 

Reagent	Final concentration	Quantity
Acrylamide	4.95%	0.33 mL
Tris 1 M, pH 6.8	125 mM	0.250 mL
10% SDS	0.1%	20 µL
10% APS	0.1%	20 µL
TEMED	0.01%	2 µL
H_2_O	n/a	1.2 mL
Total	n/a	2 mL
15. **Running buffer 10×**
  **Table:** 

Reagent	Final concentration	Quantity
Tris	25 mM	33 g
Glycine	1.92 M	144 g
SDS	1%	10 g
H_2_O	n/a	1,000 mL
Total	n/a	1,000 mL
16. **Loading buffer 5×**
  **Table:** 

Reagent	Final concentration	Quantity
Tris HCl 1 M pH 6.8	250 mM	5 mL
Glycerol	50%	10 mL
SDS	10%	2 g
Bromophenol blue 1%	0.125%	250 µL
H_2_O	n/a	4.75 mL
Total	n/a	10 mL
17. **Coomassie Blue**
  **Table:** 

Reagent	Final concentration	Quantity
Ethanol	50%	125 mL
Acetic acid	10%	25 mL
CBB R-250	0.25%	625 g
H_2_O	n/a	100 mL
Total	n/a	200 mL
  *Note 6
18. **Fast Coomassie de-staining**
  **Table:** 

Reagent	Final concentration	Quantity
Ethanol	10%	10 mL
Acetic acid	20%	20 mL
H_2_O	n/a	70 mL
Total	n/a	100 mL
19. **Coomassie de-staining**
  **Table:** 

Reagent	Final concentration	Quantity
Acetic acid	10%	10 mL
H_2_O	n/a	90 mL
Total	n/a	100 mL
20. **Ampicillin 100 µg/mL**
  **Table:** 

Reagent	Final concentration	Quantity
Ampicillin	0.1 g/mL	1 g
H_2_O	n/a	10 mL

**Laboratory supplies**

1. Standard dialysis tubing with 2000 MW cut off (e.g., Membra-Cel, Viskase, catalog number: 300911011)
2. Filters, 0.2 µm diameter (SARSTEDT, catalog number: 83.1826.001)
3. Protein concentrator (Amicon R-Ultra, 15 mL, 3 KDa) (Millipore, Merck, catalog number: UFC9003)
4. Magnetic stirrer (Fisherbrand, Fisher Scientific, catalog number: 11808892)
5. Small columns (1–5 mL bed volume) (empty PD-10 column for gravity flow purification) (Cytiva, catalog number: 17043501)
6. Electrophoresis chamber (Mini-PROTEAN Tetra Vertical Electrophoresis Cell) (Bio-Rad, catalog number: 1658004)
7. Handcast electrophoresis gel accessories (for 1.00 mm thickness gels) (Bio-Rad, catalog number: 1658001FC)
8. Microtubes, 1.5 mL (Eppendorf, catalog number: 0030120086)
9. Centrifuge tube, 50 mL (Avantor, VWR, catalog number: 525-0634)

## Equipment

1. Orbital incubator (Sartorius, model: Certomat BS-1, catalog number: 20444445202)
2. Spectrophotometer (VWR, model: V-1200, catalog number: 634-60009
3. Balance (Fisher Scientific, model: FPOS622, catalog number: 8344272595)
4. Hot plate stirrer (Fisher Scientific, model: AREX, catalog number: 15369664)
5. pH meter (pH and ORP table-top bench meter laboratory Tester Hanna) (Servovendi, catalog number: 1965)
6. Centrifuge with fixed-angle rotor (Beckman, model: Avanti J-20 XP, catalog number: 8043-30-1171)
7. Rotor JA14 (Beckman, catalog number: 339247)
8. Microcentrifuge (Eppendorf, model: 5430R, catalog number: 5428000210)
9. Eppendorf^®^ rotor F-35-6-30 (Eppendorf, Merck, catalog number: EP5427716009)
10. Eppendorf^®^ rotor F-45-48-11 (Eppendorf, Merck, catalog number: EP5427755004)
11. Spectro fluorimeter (we used an SLM-Aminco 8100 Series 2, not commercially available)
12. Quartz cuvette with two transparent faces (we used 3 mm light path, 100 μL volume) (Hellma, catalog number: 105-251-15-40)

## Software and datasets

1. Sigmaplot 11.0 (Systat Software, Inc; 2008) (any other scientific data analysis and graphing software can be used)
2. Maxchelator (https://somapp.ucdmc.ucdavis.edu/pharmacology/bers/maxchelator/CaEGTA-TS.htm)

## Procedure

- **CaM expression and purification**
  The human CaM gene, inserted into the pET-14b expression vector, is introduced into BL21-DE3 *E. coli* (other bacteria strain to express non-toxic heterologous genes can also be used). The purification procedure for CaM has been adapted from existing literature [11] and results in substantial yields of soluble protein, as outlined below.
  1. Protein expression
    - Cultivate BL21-DE3 cells from glycerol stock in 1 L of LB medium at 37 °C, supplemented with 100 μg/mL ampicillin, until the optical density (A_600_) reaches 0.8–1.
    - Induce protein expression by adding 0.4 mM IPTG and continue cultivation for 4–6 h at 37 °C or overnight at 20 °C.
  2. Cell harvesting and resuspension
    - Centrifuge the cells to collect them (9,000× *g* for 9 min at 4 °C) and wash the cell pellet twice with 50 mL of fresh lysis buffer.
    - Resuspend the cell pellet in 30 mL of lysis buffer and store the sample in 10 mL aliquots at -20 °C.
  3. Sample preparation
    - Thaw an aliquot on ice and perform sonication (three cycles of 10 s at 50 kHz; keep the sample on ice).
    - Subject the sample to three freeze–thaw cycles by alternating between a dry ice ethanol bath and a 37 °C water bath.
    - Centrifuge the sample in a microcentrifuge at 14,000× *g* for 15 min.
    - Heat the supernatant to 95 °C for 5 min, followed by centrifugation as previously described. This step leverages CaM's enhanced thermal stability.
  4. Chromatography
    - Introduce CaCl_2_ to the supernatant (final concentration: 5 mM). Load the sample at room temperature onto a 5 mL Phenyl–Sepharose column pre-equilibrated with CQ buffer. The chromatography can be achieved through gravity flow or by utilizing a peristaltic pump. Wash the column with 20 column volumes of CW buffer followed by 10 column volumes of CHSW buffer.
    - Elute CaM with 20 column volumes of CE buffer, taking fractions of 500 µL.
  5. Analysis and storage
    - Mix 20 µL of CaM with 5 µL of 5× loading buffer. Load the mixture onto a 15% acrylamide gel and perform electrophoresis using a 1× dilution of the running buffer. Run the gel at a voltage of approximately 120–150 V for 90 min. Stain the gel with Coomassie Blue for 10 min and destain first using fast destaining for 15 min followed by regular destaining for 30 min. Concentrate the sample to have a final concentration of at least 1 mg/mL using an Amicon centrifugal filter of 3 kDa. Dialyze the fractions against MilliQ water,
    - Store the purified CaM at -20 °C at 1 mg/mL in fractions of 1 mL or lyophilize it in fractions of 1 mL.
    - CaM concentration estimation can be done via absorbance at 276 nm, with ε_276_ = 3,030 M^-1^·cm^-1^. Alternatively, employ the Bradford method for quantification.
- **Dansylation of calmodulin**
  1. CaM preparation
    Begin by diluting CaM in D buffer to achieve a final concentration of 1 mg/mL.
  2. Dansyl chloride preparation
    - Dissolve dansyl chloride in acetone at a concentration of 2.17 mg/mL.
    - Store this dansyl chloride solution at either 4 °C or -20 °C in a dark environment. It remains stable for an extended period, often several months.
  3. Dansylation process
    - Add 12.5 μL of the prepared dansyl chloride solution to 1 mL of the CaM solution at 1 mg/mL. This results in a final concentration of dansyl chloride of approximately 100 μM.
    - Incubate the mixture at room temperature, in darkness, for 2 h. During this time, gently vortex the mixture every 20 min.
  4. Separation of dansylated CaM (D-CaM)
    - To separate D-CaM from any unreacted dansyl chloride, you will need a disposable column packed with approximately 1 mL of Sephadex G-25.
    - Equilibrate approximately 250 mg of dry resin with distilled water.
    - Load the D-CaM mixture onto the column and collect fractions of 50–100 μL each.
    - The initial fractions eluted, known as the "excluded fraction," contain the D-CaM conjugate.
    See Note 7.
    To determine the specific dansylated residues in CaM, tryptic digestion coupled with mass spectroscopy or gas-phase protein sequencers have been employed, reporting dansylation at either Lys75 or Lys 115 [12,13]. The mass spectrometry analysis revealed the binding of up to four dansyl molecules per CaM. Furthermore, tandem mass spectrometry of tryptic peptides strongly indicates dansylation at Ala1 and Lys148 [14]. The identification of the remaining two dansylated residues is pending further investigation; however, the data are consistent with the possibility of them being Lys75 and Lys115 [14].
  5. Fraction analysis
    Swiftly verify the presence of the protein in the collected fractions by performing dot blotting on nitrocellulose and staining it with Ponceau Red. Additionally, analyze the fractions using 15% SDS-PAGE gels. For a more detailed examination, record the emission spectra of each sample (as further explained below).
  6. D-CaM storage
    - Gather the fractions containing D-CaM and concentrate them if needed. We recommend stocks between 0.5 and 5 µM.
    - Store the resulting D-CaM aliquots in a dark environment at -20 °C or, alternatively, as lyophilized samples. Typically, the conjugate retains its properties when stored at -20 °C for several months or more.
    See Note 8.
  7. Protein concentration determination
    Utilize the Bradford assay to determine the protein concentrations of D-CaM, using unlabeled CaM as a standard. Alternatively, measure the concentration of D-CaM by UV absorption at 320 nm (ε_320_ = 3,400 M^-1^·cm^-1^).
    Dansyl moiety concentration:
    Determine the concentration of the incorporated dansyl moiety via spectroscopy.
    When possible, calculate the number of specific dansylated residues within a D-CaM molecule.
    This process allows for the efficient dansylation of CaM, making it ready for various downstream applications and analyses.
- **Peptide resuspension**
  When working with peptides, start by dissolving them in distilled, sterile water. This is especially suitable for short peptides (<5 residues). For each specific peptide, choose the most appropriate conditions to ensure optimum solubility based on its sequence.
  1. Calculate overall charge; begin by assessing the overall charge of the peptide:
    Assign a value of -1 for each acidic residue, including aspartic acid (Asp or D), glutamic acid (Glu or E), and the C-terminal -COOH.
    Assign a value of +1 for each basic residue, including arginine (Arg or R), lysine (Lys or K), histidine (His or H), and the N-terminal -NH2. Calculate the net charge of the peptide.
    - Positive charge peptides; if the overall charge of the peptide is positive:
      Attempt to dissolve the peptide in water initially. If water does not work, try a 10%–30% acetic acid solution. If the peptide still does not dissolve, add a small amount of TFA (<50 μL) to solubilize it and then dilute to the desired concentration.
    - Negative charge peptides; if the overall charge of the peptide is negative:
      Start by attempting to dissolve the peptide in water. If water is ineffective to dissolve the peptide, add a small amount of NH_4_OH (<50 μL) and dilute to the desired concentration. See Note 9.
    - Neutral charge peptides; for peptides with a net charge of zero:
      Introduce organic solvents as follows: First, try adding acetonitrile, methanol, or isopropanol. For highly hydrophobic peptides, start with a small amount of DMSO (30–50 μL, 100%). Gradually add this solution drop by drop to a stirring aqueous buffered solution like PBS or your preferred buffer until the desired peptide concentration is achieved. If turbidity appears in the peptide solution, it indicates that you have reached the limit of solubility. In such cases, sonication can help to dissolve the peptides. If the peptide includes cysteine residues, use DMF instead of DMSO. See Note 10.
      In cases where peptides tend to aggregate, incorporate 6 M guanidine•HCl or 8 M urea before proceeding with necessary dilutions.
- **Peptide interaction with Apo-CaM**
  1. Dilute the peptide stock in fluorescence buffer to have a 20 μM peptide solution.
  2. Calculate the volumes of peptide required for each titration step using the formula: C_1_·V_1_ = C_2_·V_2_, where C_1_ is the initial peptide concentration in the stock (20 µM), V_1_ is the volume of peptide to add in each step, C_2_ is the desired final peptide concentration in each step, and V_2_ is the total sample volume (100 µL).
    Prepare a table with the calculated volumes (Table 1):
    **Table 1.** Sample composition for peptide titration in absence of CaCl_2_

Peptide final concentration (µM)	D-CaM 1 µM (µL)	Peptide 20 µM (µL)	Fluorescence buffer volume (µL)
0	5	0	95
0.1	5	0.5	94.5
0.2	5	1	94
0.5	5	2.5	92.5
1	5	5	90
1.5	5	7.5	87.5
2	5	10	85
4	5	20	75
8	5	40	55
16	5	80	15
  3. Following sample preparation, centrifuge them at 185,494× *g* (radius 98 mm) for 10 min and carefully transfer the resulting supernatants to fresh 500 µL tubes to remove any potential aggregates.
    Ensure the absence of air bubbles as they can distort fluorescence readings. Insert the cuvette into the fluorimeter and proceed to acquire emission spectra. Employ an excitation wavelength of 340 nm, recording emissions across the 400–660 nm range. All measurements are conducted at a temperature of 25 °C, with necessary corrections applied to account for any buffer-related interference. These conditions should yield a prominent fluorescence peak around 500 nm in the D-CaM spectrum. Record the fluorescence values for each peptide concentration.
  4. Analyze data to determine the interaction between the peptide and D-CaM at different concentrations.
**Calcium titration:**
  1. Start the process by gradually adding concentrated CaCl_2_ aliquots into a cuvette containing the peptide saturation sample once the peptide concentration for signal saturation is achieved.
  2. In a 100 μL sample of the peptide saturation sample, add 1 μL of Ca buffer solution sequentially. It is important to note that all experiments will be conducted under constant conditions of pH 7.4 and 25 °C. Remember that EGTA buffering is pH dependent.
    It is worth emphasizing that the experimental design incorporates a 10 mM Ca buffer, affording the evaluation of free calcium concentrations spanning from nM to µM ranges. Notably, adjustments in CaCl_2_ concentration are permissible, and alternative buffers may be employed to facilitate analyses across diverse calcium concentration ranges, whether elevated or diminished.
  3. Utilize the Ca-EGTA Calculator v1.3, incorporating constants sourced from Theo Schoenmakers' Chelator, particularly Table 2, which corresponds to the 10 mM CaCl_2_ calcium buffer. After each Ca^2+^ addition, ensure thorough mixing to attain homogeneity.
    See Note 11.
    **Table 2.** Free calcium concentration calculation for 10 mM CaCl_2_ solution

Volume of Ca Buffer (μL)	Total volume (μL)	[Ca^2+^] total (mM)	[Ca^2+^] free (µM)
0	100	0	0
1	101	0.1	0.0030
2	102	0.2	0.0079
3	103	0.3	0.177
4	104	0.4	0.471
5	105	0.5	2.45
6	106	0.6	10.01
7	107	0.7	20
8	108	0.8	30
9	109	0.9	40
10	110	1.0	50
  4. Record the fluorescence spectra approximately 20–30 s after adding each sample to the cuvette. Note that extended equilibration times do not yield improved data.
  5. Continue the titration of Ca^2+^ until saturation is reached, signified by the absence of further observable changes in the spectra.
- **Peptide interaction with Holo-CaM**
  1. To evaluate the peptide's interaction with holo-D-CaM, first saturate CaM with 1 mM free CaCl_2_.
    Calculate the necessary peptide volumes for each titration step using the equation: C_1_·V_1_ = C_2_·V_2_, where C_1_ represents the initial peptide concentration in the stock (20 μM), V_1_ is the volume of peptide to be added in each step, C_2_ is the desired final peptide concentration for each step, and V_2_ is the total sample volume (100 μL).
    Create a table to document the calculated volumes (Table 3):
    **Table 3.** Sample composition for peptide titration in the presence of 1 mM CaCl_2_

Final peptide concentration (µM)	Peptide 20 µM volume (µL)	F buffer (µL)	Ca buffer (µL)	Total V (µL)
0	0	90	10	100
0.2	1	89	10	100
0.5	2.5	87.5	10	100
1	5	85	10	100
2	10	90	10	100
4	20	80	10	100
8	40	50	10	100
16	80	10	10	100
  2. After preparing the samples, centrifuge them at 185,494× *g* (radius 98 mm) for 10 min and carefully transfer the resulting supernatants to fresh 500 μL tubes to eliminate any potential aggregates.
  3. Ensure the absence of air bubbles, as they can distort fluorescence measurements. Insert the cuvette into the fluorimeter and proceed to acquire emission spectra. Use an excitation wavelength of 340 nm, recording emissions across the 400–660 nm range. All measurements are conducted at a temperature of 25 °C, with appropriate corrections made to account for any buffer-related interference. These conditions should yield a prominent fluorescence peak around 500 nm in the D-CaM spectrum. Record the fluorescence values for each peptide concentration.

## Data analysis

**Figure 1.**
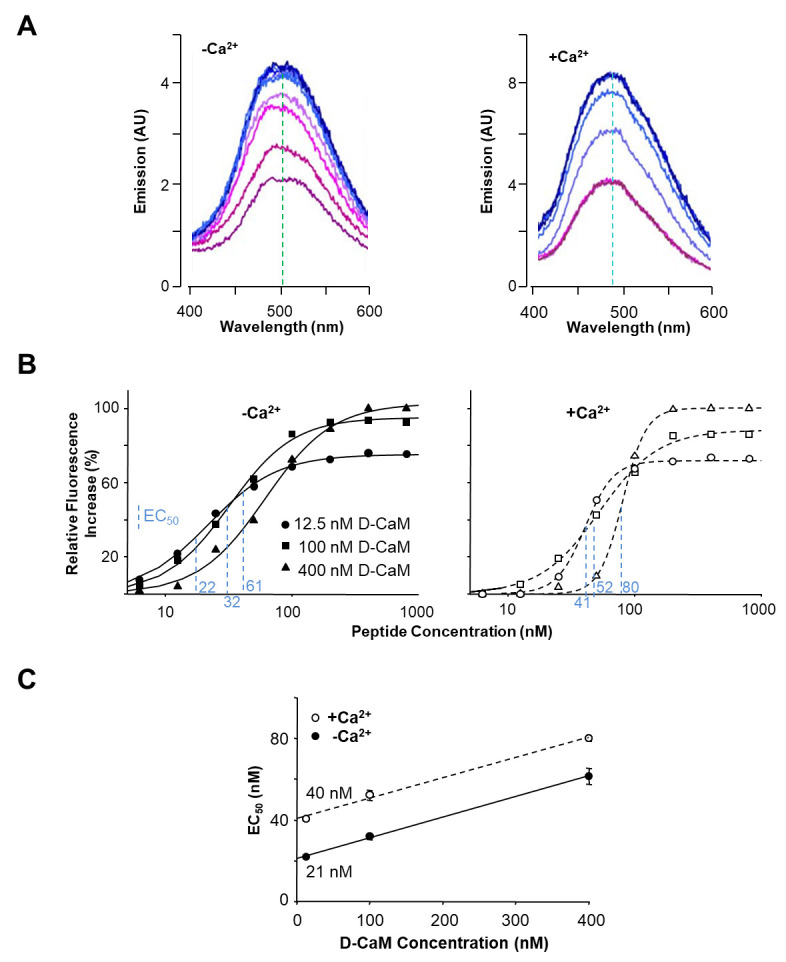
Data analysis from emission spectra. A. Effect of incremental concentrations of a peptide in the dansyl-CaM (D-CaM) fluorescence emission spectrum in absence (left) and presence (right) of Ca^2+^. The wavelength at which the maximal emission is achieved is indicated by the vertical discontinuous line (adapted from Alaimo et al. [15]). B. Relative concentration-dependent enhancement of fluorescence at varying peptide concentrations using fixed 12.5, 100, and 400 nM D-CaM concentrations, in absence (left) and presence (right) of Ca^2+^. EC_50_ values are indicated in blue for each condition. C. Extrapolation to the true affinity. Plot of the concentration of the peptide at which 50% of maximal response is achieved (EC_50_) at different D-CaM concentrations (adapted from Bonache et al. [16]).

In evaluating the affinity of a specific peptide for D-CaM, our approach involves the analysis of emission spectra obtained at various peptide concentrations, depicted in Figure 1A. Notably, a correlation is observed between peptide concentration and the intensity of the emission spectrum. The emission spectra exhibit a discernible trend until reaching a saturation point, wherein further increments in peptide concentration no longer elicit changes in the maximal emission intensity.

To better assess changes in intensity, we focus on the wavelength range of 490–500 nm (Table 4). We quantify intensity increases by summing up the values within this range. For normalization, we use the apo-CaM condition as the baseline, ensuring that D-CaM with 0 μM peptide corresponds to an intensity of 0. To normalize, we divide the sum of intensities by the corresponding value obtained in the apo-CaM condition (Table 4).

The experimental protocol is subsequently reiterated under Holo-CaM conditions, wherein a saturating concentration of unbound Ca^2+^ (e.g., 10 mM) is initially introduced, followed by the repetition of the peptide titration process.

For a more lucid depiction of intensity changes, we present the normalized values as percentages, setting the maximum intensity as 100. This normalization entails dividing each value by the maximum intensity obtained and then multiplying the result by 100 (Table 1).

**Table 4.** Experimental data summary. The values in the *Normalized data* column are obtained by dividing each intensity value by the corresponding value obtained in apoD-CaM and subtracting 1. The *Percentage* column represents the percentages, with the maximum taken as 100%, calculated by dividing all values by the maximum normalized value.

Peptide [μM]	Sum of intensity (490–500 nm)	Normalized data (relative to apoD-CaM)	Percentage
0	x1	(x1/x1_apoD-CaM) - 1 = y1	(y1/y*max*) * 100
2	x2	(x2/x1_apoD-CaM) - 1 = y2	(y2/y*max*) * 100
4	x3	(x3/x1_apoD-CaM) - 1 = y3	(y3/y*max*) * 100
...	...	...	...
n	x_*max*	(x_*max*/x_1_apoD-CaM) – 1 = y*max*	(y*max*/y*max*)*100 = 100

If we plot the obtained values against the peptide concentration, a dose-response curve in percentage is generated. This approach facilitates a robust comparison of intensity changes across various peptide concentrations, culminating in a percentage-based representation of the specified peptide's affinity for D-CaM.

To generate concentration-response curves, plot fluorescence enhancement against the peptide-D-CaM ratio or [peptide] and fit the data using the three-parameter Hill equation through curvilinear regression.

EC_50_ values vary with D-CaM concentration due to ligand depletion, especially at low concentrations. Correct for depletion by determining EC_50_ values across a range of D-CaM concentrations. At infinitely low D-CaM concentrations, depletion should be negligible, making EC_50_ a true affinity value [1]. For accurate dissociation constant (K_d_) determination, perform titrations with varying initial concentrations of D-CaM (6.25–200 nM) and create concentration-response curves at each D-CaM concentration (Figure 1B). Calculate apparent dissociation constants (EC_50_) from Figure 1B and plot them against D-CaM concentrations (Figure 1C). Obtain true dissociation constants through linear fitting and extrapolation to D-CaM concentrations equal to zero.Principio del formulario

If stoichiometry is known, estimate K_d_ values using a Scatchard plot analysis. However, we recommend the previous method for its accuracy and reliability in K_d_ determination, particularly in complex binding interactions.

To estimate K_d_, assuming a 1:1 stoichiometry, apply the equation:

$F=F_{max\;}$ × $\;(1-\frac{2\cdot K_{d}}{\left[peptide\right]-\left[CaM\right]+\;K_{d}+\;\sqrt{\left(\left[peptide\right]-\left[CaM\right]-K_{d}{)}^{2}+4\cdot K_{d}\cdot [peptide\right]}}$

Here, F represents the increase in fluorescence, F_max_ is the maximal fluorescence (variable), [peptide] is the known total peptide concentration, [CaM] is the known concentration of total D-CaM, and K_d_ is the variable for the affinity constant.

Express results as means ± S.E.M from three or more experiments. For statistical analysis, use the unpaired Student t-test, with P < 0.05 (*), P < 0.01 (**), and P < 0.001 (***) considered statistically significant. This protocol provides a comprehensive method for estimating the K_d_ in a fluorescence-based ligand displacement assay with D-CaM, considering the impact of D-CaM concentration and correcting for ligand depletion at low concentrations. It offers a robust approach for characterizing the binding affinity and cooperativity between D-CaM and the ligand.

## Validation of protocol

This methodology, initially validated by Kincaid and Vaughan in 1986 [17], has exhibited consistent efficacy in discerning conformational changes resulting from interactions with Ca^2+^, peptides, or proteins. Subsequent studies Yuan by and Graves in 1989 [18] investigated the interaction of CaM with the γ subunit of phosphorylase kinase, while Munier et al. in 1991 [19] characterized the catalytic and calmodulin-binding domains of *Bordetella pertussis* adenylate cyclase (refer to Figures 3 and 4). Filoteo et al. [20] delved into the binding between the lipid-binding region (G region) of the erythrocyte Ca^2+^ pump and CaM (see Figures 4 and 5). In 2013, Alaimo et al. [14] explored the interaction of CaM with different components of the Kv7.2 channel (refer to Figures 5, 6, and 7). Zhang et al. [21] identified two distinct CaM binding sites in the angiotensin II (AT1A) receptor (refer to Figures 4 and 6). Alcalde et al. [22] demonstrated that non-myristoylated peptides derived from the CaM binding site of Grb7 exhibit higher efficiency in binding dansyl-CaM in the presence of Ca^2+^ compared to its absence (see Supplementary Figure 2). More recently, Nuñez et al. [23] showcased that the oxidation of peptides derived from the S2S3 linker of Kv7 channels inhibits their binding to CaM (refer to Figure 3), and numerous other studies contribute to the comprehensive utility of this method.

## General notes and troubleshooting

1. We request peptides with a purity level exceeding 90%, which, alongside maintaining the correct sequence, should also possess an amino group (-NH3) at the N-terminus and a carboxyl group (-COOH) at the C-terminus. These terminal groups are instrumental in promoting the stability and suitability of the peptide for subsequent experimental procedures, such as binding assays and functional studies.
2. The initial Ca^2+^ concentration within the D-CaM sample plays a pivotal role in these assays. Consequently, it is imperative to minimize any residual Ca^2+ ^presence. To achieve this, we use 5 mM EGTA to ensure that the minimal endogenous Ca^2+^ bound to the protein under titration does not interfere with free Ca^2+^ levels.
3. Ensure the pH is promptly readjusted to 7.4 after buffer preparation, as CaCl_2_ addition can lead to pH reduction.
4. We suggest dissolving Tris in 150 mL of solution, carefully adjusting the pH to 8.8 with HCl, and then precisely adding the necessary volume to reach a final volume of 200 mL.
5. We suggest dissolving Tris in 75 mL of solution, carefully adjusting the pH to 6.8 with HCl, and then precisely adding the necessary volume to reach a final volume of 100 mL.
6. Dissolve for 2 h with continuous stirring and filter using filter paper. Store at room temperature, protected from light.
7. To eliminate the excess dansyl chloride, we recommend dialyzing the D-CaM samples in conjunction with the gel filtration process outlined earlier.
8. Collect the D-CaM fractions, concentrate if required, and safeguard them by storing in light-protected aliquots at -20 °C or by lyophilization. Our thorough investigations have demonstrated that the conjugate's properties remain largely unchanged when stored at -20 °C for months, if not longer [14].
9. Ensuring that the protein or peptide samples remain uniform without aggregation is of utmost importance. We routinely employ dynamic light scattering (DLS) with a Zetasizer Nano instrument (Malvern Instruments Ltd.) to assess sample dispersion. It is essential to use samples exhibiting monodispersity, where both the correlation function and polydispersity index are below 0.2.
10. When dealing with peptides that contain cysteine (Cys) residues, it is advisable to abstain from employing basic solutions for dissolution and explore alternative methods. In such cases, ideal solvents include degassed mediums like low-pH buffers, diluted acetic acid, or a solution consisting of 0.1% trifluoroacetic acid in aqueous acetonitrile. Particular caution should be exercised to avoid the use of DMSO, especially when working with peptide trifluoroacetates.
11. The key to achieving accurate Ca^2+^ titrations is the precise control of free Ca^2+^ levels using chelators like EGTA or EDTA. We prefer EGTA for experiments conducted at pH 7.5 to replicate intracellular conditions and physiological magnesium concentrations. Maintaining strict pH control is vital because chelating efficiency is highly sensitive to proton concentration. To calculate free Ca^2+^ concentrations, we employ the Maxchelator program (http://maxchelator.stanford.es) and custom software. Various other programs are available to determine free Ca^2+^ concentrations based on the total added Ca^2+^, given EGTA levels, and considering different temperature, ionic strength, and pH conditions.

## References

[1] Chin D. and Means A. R. (2000). Calmodulin: a prototypical calcium sensor. Trends Cell Biol. 10(8): 322-328. 10884684 10.1016/s0962-8924(00)01800-6

[2] Rhoads A. R. and Friedberg F. (1997). Sequence motifs for calmodulin recognition. FASEB J. 11(5): 331-340. 9141499 10.1096/fasebj.11.5.9141499

[3] Rasmussen C. D., Means A. R. and Norris R. E. (1983). Interaction of cyclic nucleotide phosphodiesterase with calmodulin and its subunits. J. Biol. Chem. 258(9): 6016-6020.

[4] Klee C. B. and Vanaman T. C. (1982). Calmodulin. Adv. Protein Chem. 35: 213-321. 6762067 10.1016/s0065-3233(08)60470-2

[5] Walker J. M. (1984). The Bovine Adrenal Chromaffin Cell: A Model for Studies of Calcium, Protein, and Secretion. Biochemical Education 12(4): 170-175.

[6] Hawe A., Sutter M. and Jiskoot W. (2008). Extrinsic Fluorescent Dyes as Tools for Protein Characterization. Pharm. Res. 25(7): 1487-1499. 18172579 10.1007/s11095-007-9516-9 PMC2440933

[7] O'Neil K. T. and DeGrado W. F. (1990). How calmodulin binds its targets: sequence independent recognition of amphiphilic α-helices. Trends Biochem. Sci 15(2): 59-64. 2186516 10.1016/0968-0004(90)90177-d

[8] Chen X., Xing J., Jiang L., Qian W., Wang Y., Sun H., Wang Y., Xiao H., Wang J., Zhang J., .(2016). Involvement of calcium/calmodulin-dependent protein kinase II in methamphetamine-induced neural damage. J. Appl. Toxicol. 36(11): 1460-1467. 26923100 10.1002/jat.3301

[9] Peersen O. B., Madsen T. S. and Falke J. J. (1997). Intermolecular tuning of calmodulin by target peptides and proteins: Differential effects on Ca^2+^ binding and implications for kinase activation. Protein Sci. 6(4): 794-807. 9098889 10.1002/pro.5560060406 PMC2144748

[10] Smith L. D., Jones D. P. and Curnutte J. T. (2005). Studies on the extrinsic fluorophores of proteins. J. Biol. Chem. 280(16): 15952-15960.15713671

[11] Vogel H. J., Lindahl L. and Thulin E. (1983). Calcium dependent hydrophobic interaction chromatography of calmodulin, troponin C and their proteolytic fragments. FEBS Lett 157: 241-246.

[12] Mori M., Konno T., Ozawa T., Murata M., Imoto K. and Nagayama K. (2000). Novel interaction of the voltage-dependent sodium channel(VDSC) with calmodulin: does VDSC acquire calmodulin-mediated Ca^2+^-sensitivity. Biochemistry 39: 1316-1323. 10684611 10.1021/bi9912600

[13] Torok K., Cowley D. J., Brandmeier B. D., Howell S., Aitken A. and Trentham D. R. (1998). Inhibition of calmodulin-activated smooth-muscle myosin light-chain kinase by calmodulin-binding peptides and fluorescent(phosphodiesterase activating) calmodulin derivatives. Biochemistry 37: 6188-6198. 9558358 10.1021/bi972773e

[14] Alaimo A., Malo C., Areso P., Aloria K., Millet O. and Villarroel A. (2013) The use of dansyl-calmodulin to study interactions with channels and other proteins. Methods Mol. Biol. 998: 217-231. 23529433 10.1007/978-1-62703-351-0_17

[15] Alaimo A., Alberdi A., Gomis-Perez C., Fernández-Orth J., Bernardo-Seisdedos G., Malo C., Millet O., Areso P. and Villarroel A. (2014). Pivoting between calmodulin lobes triggered by calcium in the Kv7.2/calmodulin complex. PLoS One 9(1): e86711. 24489773 10.1371/journal.pone.0086711 PMC3904923

[16] Bonache M.A., Alaimo A., Malo C., Millet O., Villarroel A. and González-Muñiz R. (2014). Clicked bis-PEG-Peptide Conjugated for Studying Calmodulin-Kv7.2 Channel Binding. Org. Biomol. Chem. 12: 8877-8887. 25264745 10.1039/c4ob01338g

[17] Kincaid R. L. and Vaughan M. (1986). Direct comparison of Ca^2+^ requirements for calmodulin interaction with and activation of protein phosphatase. Proc Natl Acad Sci USA. 83(5):1193-1197. 3006040 10.1073/pnas.83.5.1193 PMC323041

[18] Yuan C. J. and Graves D. J. (1989). Ca^2+^-independent interaction of the gamma subunit of phosphorylase kinase with dansyl-calmodulin. Arch Biochem Biophys. 274(2): 317-326. 2508559 10.1016/0003-9861(89)90445-1

[19] Munier H., Gilles A.-M., Glaser P., Krin E., Danchin A., Sarfati R. and Barzu O. (1991). Isolation and characterization of catalytic and calmodulin-binding domains of *Bordetella pertussis* adenylate cyclase. Eur. J. Biochem. 196: 469-474. 2007407 10.1111/j.1432-1033.1991.tb15838.x

[20] Filoteo A. G., Enyedi A. and Penniston J. T. (1992). The lipid-binding peptide from the plasma membrane Ca^2+^ pump binds calmodulin, and the primary calmodulin-binding domain interacts with lipid. J. Biol. Chem. 267(17): 11800-11805. 1318301

[21] Zhang R., Liu Z., Qu Y., Xu Y. and Yang Q. (2013). Two Distinct Calmodulin Binding Sites in the Third Intracellular Loop and Carboxyl Tail of Angiotensin II(AT_1A_) Receptor. PLOS ONE 8(6): e65266. 23755207 10.1371/journal.pone.0065266 PMC3673938

[22] Alcalde J., González-Muñoz M. and Villalobo A. (2020). Grb7-derived calmodulin-binding peptides inhibit proliferation, migration and invasiveness of tumor cells while they enhance attachment to the substrate. Heliyon 6(5): e03922. 32420488 10.1016/j.heliyon.2020.e03922 PMC7215194

[23] Nuñez E., Jones F., Muguruza-Montero A., Urrutia J., Aguado A., Malo C., Bernardo-Seisdedos G., Domene C., Millet O., Gamper N. and Villarroel A. (2023). Redox regulation of KV7 channels through EF3 hand of calmodulin. eLife 12: e81961. 36803414 10.7554/eLife.81961 PMC9988260

